# Pre-service Science Teachers’ Neuroscience Literacy: Neuromyths and a Professional Understanding of Learning and Memory

**DOI:** 10.3389/fnhum.2019.00020

**Published:** 2019-02-14

**Authors:** Finja Grospietsch, Jürgen Mayer

**Affiliations:** Department of Biology Education, University of Kassel, Kassel, Germany

**Keywords:** neuromyths, pre-service science teachers, neuroscience literacy, professional knowledge, beliefs, misconceptions, teaching profession, learning and memory

## Abstract

Transferring current research findings on the topic of learning and memory to “brain-based” learning in schools is of great interest among teachers. However, numerous international studies demonstrate that both pre-service and in-service teachers do not always succeed. Instead, they transfer numerous misconceptions about neuroscience, known as neuromyths, into pedagogical practice. As a result, researchers call for more neuroscience in teacher education in order to create a professional understanding of learning and memory. German pre-service science teachers specializing in biology complete neuroscientific modules (*human biology*/*animal physiology*) during their studies because they are expected to teach these topics to their students. Thus, they are required to demonstrate a certain degree of neuroscience literacy. In the present study, 550 pre-service science teachers were surveyed on neuromyths and scientific concepts about learning and memory. Pre-service science teachers’ scientific concepts increased over the course of their training. However, beliefs in neuromyths were independent of participants’ status within teacher education (first-year students, advanced students, and post-graduate trainees). The results showed that 10 neuromyths were endorsed by more than 50% of prospective science teachers. Beliefs in the existence of learning styles (93%) and the effectiveness of Brain Gym (92%) were most widespread. Many myths were endorsed even though a large share of respondents had thematically similar scientific concepts; endorsement of neuromyths was found to be largely independent of professional knowledge as well as theory-based and biography-based learning beliefs about neuroscience and learning. Our results suggest that neuromyths can exist in parallel to scientific concepts, professional knowledge and beliefs and are resistant to formal education. From the perspective of conceptual change theory, they thus exhibit characteristic traits of misconceptions that cannot simply be counteracted with increased neuroscientific knowledge. On the basis of our study’s findings, it can be concluded that new teacher programs considering neuromyths as change-resistant misconceptions are needed to professionalize pre-service science teachers’ neuroscience literacy. For this, an intensive web of exchange between the education field and neuroscientists is required, not just to deploy the latest scientific insights to refute neuromyths on learning and memory, but also to identify further neuromyths.

## Introduction

Findings from brain research have unleashed a veritable “neuro-boom” in recent years, which has taken the form of numerous publications for teachers as well as learning guides for students (e.g., Doyle and Zakrajsek, 2013). Teachers in all subjects have expressed great interest in neuroscience research findings and find it useful to incorporate them into their instruction (Dekker et al., 2012). Even incorrectly interpreted research findings have great appeal once images of the brain and/or neuroscientific explanations are added (McCabe and Castel, 2008; Lindell and Kidd, 2013). Media and even educational programs make use of this effect; they are filled with bold and eye-catching yet empty promises like ‘learn while you sleep’ or ‘innate intelligence through Brain Gym’. Money, time, and effort are expended integrating so-called “neuromyths” into the school system (Pasquinelli, 2012). It is understandable that people who lack knowledge in the field of neuroscience might struggle to distinguish facts from myths (Wallace, 1993; Beck, 2010). However, even teachers, the alleged experts on learning, endorse misconceptions^1^ about neuroscience and base their pedagogical practice on neuromyths (e.g., Dekker et al., 2012).

The term “neuromyth” was coined in the 1980s by the neurosurgeon Alan Crockard, who used it to refer to unscientific understandings of the brain in medical culture (Howard-Jones, 2010). The Organization for Economic Co-operation and Development [OECD] (2002) defines neuromyths as “misconception[s] generated by a misunderstanding, a misreading, or a misquoting of facts scientifically established (by brain research) to make a case for use of brain research in education and other contexts” (p. 111). Neuromyths are thus falsely or overly interpreted neuroscientific research findings that are transferred to applied contexts such as teaching, learning and instruction. Neuromyths are often seen as originating in simplistic language in the reporting of neuroscientific research findings. These research findings are often published at a challenging reading level (Yeung et al., 2018) and tend to be very complex and difficult for non-neurobiologists to understand, meaning that simplistic formulations are often resorted to. These ‘pop-science’ statements are then falsely interpreted and quickly lose their kernel of truth. They are packed into “low-cost and easily implemented classroom approaches” (Howard-Jones, 2014, p. 819) that claim to promote learning. While fun, these approaches also result in the rapid propagation of neuromyths among students, parents, and teachers. This process is strengthened by media, whose simplified and/or overly interpreted portrayals of research findings reach a wide audience (Wallace, 1993; Beck, 2010), and companies looking to offer learning programs that claim to be “brain-based” but usually provide consumers with little hope of learning success (Goswami, 2006; Pasquinelli, 2012). The OECD has been calling attention to the problem of neuromyths almost as long as it has been calling for teaching and learning to be based on neuroscience.

Neuromyths often originate from overgeneralizations of empirical research (Macdonald et al., 2017). Today, neuromyths have emerged for many aspects of neuroscience, including specific learning difficulties such as dyslexia (Macdonald et al., 2017) and the influence of nutrition (Dekker et al., 2012) or music (Düvel et al., 2017) on the brain. This study focuses on neuromyths related to learning and memory. Table 1 illustrates with three examples how these neuromyths arise from errors in transferring neuroscientific information (the kernel of truth). The depicted transfer steps (left) as well as their relationships to neuroscientific findings (right) are based on a summary of the current state of theory on neuromyths as well as supplementary literature research.

**Table 1:** Errors in argumentation from the neuroscientific kernel of truth to erroneous implications for school instruction compared to the neuroscientific evidence.

	Neuromyth fallacies	Neuroscientific evidence
**Learning while you sleep** (fallacies described on the basis of Centre for Educational Research) and Innovation and Organization for	**Kernel of truth:** Overnight restructuring processes in the brain can allow new insights to be gained (consolidation) (Maquet, 2001; Gais and Born, 2004).	
Economic Co-operation and Development [OECD], 2007	Sleep can be used as additional learning time,…	Information is encoded while one is awake, and consolidated during sleep. Both processes are necessary to store knowledge for the long term, or in other words, to learn (Gais and Born, 2004).
	…a person can learn completely new content during sleep,…	It is impossible to learn new content during sleep (Stickgold, 2012). Encoding new information during sleep would disrupt the process of consolidating previously encoded information (Gais and Born, 2004).
	…and exposing oneself to acoustic stimuli allows sleeping time to be used for learning.	The brain is relatively strongly cut off from the outside world during sleep (Muzet, 2007), even though a person can react to sensory inputs such as smells by modifying breathing intensity for example (Stickgold, 2012), which makes conditioning possible (Arzi et al., 2012).
	Learners should use audio files (e.g., vocabulary words in a new language) while sleeping.	This is an implication for instruction for which no neuroscientific evidence exists.

**Logic in the left hemisphere, creativity in the right** (fallacies described on the basis of Geake, 2008)	**Kernel of truth:** There are two brain hemispheres that are not fully identical, either anatomically or functionally (hemispheric assymmetry) (Jäncke, 2013; Ocklenburg et al., 2014).	
	Each hemisphere works autonomously…	The hemispheres are connected via the *Corpus Callosum* (Bloom and Hynd, 2005).
	…and has a separate job. The left hemisphere is responsible for intellectual, rational, verbal and analytical thought, while the right hemisphere is responsible for creative, intuitive, and non-verbal thought processes.	Taking language as an example: The left hemisphere specializes in many but not all verbal processes. Some language components are rooted in the right hemisphere, such as speech melody or reading between the lines (Lai et al., 2015).
	Society and the school system pay too much attention to the left hemisphere and overburden one side of the brain.	Lateralization is not complete (Nielsen et al., 2013), the two hemispheres work together (Singh and O’Boyle, 2004).
	Both hemispheres should be addressed to an equal extent during learning and interactions between them should be fostered.	This is an implication for instruction for which no neuroscientific evidence exists.

**Only use 10% of the brain** (fallacies described on the basis of Geake, 2008 and Lilienfeld et al., 2010)	**Kernel of truth:** Imaging techniques can show which specific areas of the brain are involved in certain mental or physical actions (Biswal et al., 2010).	
	Only the colored areas of the brain are active…	Figures showing patterns of activity are differential images in which areas significantly exceeding a basic activity level are highlighted in color (Darvas et al., 2004).
	…other brain regions (gray-shaded areas) are totally inactive at this time.	Even gray-shaded areas are in a kind of “standby mode” that involves anticipatory activity (Whittingstall and Logothetis, 2009).
	There is a ‘silent cortex’ that does not trigger a visible physical reaction when stimulated and thus has no function…	These ostensibly ‘silent’ areas of the cerebral cortex are part of the association cortex and take on important functions related to higher psychological, psychosocial, and mental abilities (Baer et al., 2018).
	…and only 10% of our brain consist of neurons; the rest are functionless glial cells.	The ratio of glial cells to neurons is about 1:1 and glial cells perform important functions to support neurons and participate in memory formation (Hilgetag and Barbas, 2009).
	Learners‘ brain capacity must be increased.	This is an implication for instruction for which no neuroscientific evidence exists.

The existing research on neuromyths primarily focuses on teachers. Studies investigating the endorsement of neuromyths among teachers of various subjects have been conducted in the Netherlands, England (Dekker et al., 2012; Simmonds, 2014), Latin America (Bartoszeck and Bartoszeck, 2012; Gleichgerrcht et al., 2015; Hermida et al., 2016), Portugal (Rato et al., 2013), Australia (Bellert and Graham, 2013; Horvath et al., 2018), Greece (Deligiannidi and Howard-Jones, 2015), China (Pei et al., 2015), Turkey (e.g., Karakus et al., 2015), Switzerland (Tardif et al., 2015), Spain (Ferrero et al., 2016), the United States (Lethaby and Harries, 2016), and Canada (Macdonald et al., 2017). All of these studies have found that teachers believe in a large number of neuromyths, although only a few of these, such as Brain Gym, are related to the topic of learning and memory and there are country-specific differences in the endorsement of specific myths.^2^ Cultural differences between countries seem to have an influence on which neuromyths spread (Pei et al., 2015; Ferrero et al., 2016; Hermida et al., 2016).

One study of post-graduate teacher trainees found that 56–83% of respondents encountered educational programs based on neuromyths in their first year working in schools, which was associated with high levels of acceptance of those myths (Howard-Jones et al., 2009). Studies by Fuentes and Risso (2015, Spain), Dündar and Gündüz (2016, Turkey), Canbulat and Kiriktas (2017, Turkey), Düvel et al. (2017, Germany), Kim and Sankey (2017, Australia), Papadatou-Pastou et al. (2017, Greece), and Im et al. (2018, South Korea) indicate that neuromyths are already present during the academic stage of teacher education. However, no studies focusing on neuromyths related to learning and memory have been conducted with pre-service samples either. Nevertheless, it can generally be concluded on the basis of these results that the neuroscience content knowledge necessary to critically evaluate neuromyths does not seem to be integrated into teacher education to a sufficient degree (Howard-Jones, 2014).

Most studies have not found personal characteristics like age, professional experience, teaching subject, school type, school location (urban/rural), and participation in professional development trainings to be associated with endorsement of neuromyths or with scientific concepts about the brain (Dekker et al., 2012; Rato et al., 2013; Gleichgerrcht et al., 2015). Only Ferrero et al. (2016) found a correlation with gender, with female teachers more likely to endorse neuromyths. Macdonald et al. (2017) found evidence that age (being younger), training (having a university degree), and enrollment in neuroscience courses predict reduced endorsement of neuromyths. The topic of neuroscience is of great interest to teachers internationally (e.g., Dekker et al., 2012), but there seems to be a large gap between teachers’ interest and their ability to actually deal with neuroscientific findings in a professional way (Rato et al., 2013). Teachers with high levels of scientific concepts about the brain have proven to be more susceptible to neuromyths (Dekker et al., 2012; Rato et al., 2013; Gleichgerrcht et al., 2015; Ferrero et al., 2016; Düvel et al., 2017; Canbulat and Kiriktas, 2017) in almost all studies (except Howard-Jones et al., 2009). Horvath et al. (2018) found the acceptance of neuromyths to be nearly identical between populations of award-winning and non-award-winning teachers. There seems to be a general tendency to agree with neuroscientific statements, but a lack of ability to separate myths from facts (Ferrero et al., 2016). While reading scientific articles can reduce endorsement of neuromyths, teachers tend to use pop-science sources like TV and the Internet as their main sources of information for neuroscientific facts (Rato et al., 2013).

Im et al. (2018) demonstrated that taking an educational psychology course only improves neuroscience literacy; it does not reduce beliefs in neuromyths. Macdonald et al. (2017) found differences in endorsement of neuromyths between the general public, teachers, and people with high levels of neuroscientific knowledge, and Papadatou-Pastou et al. (2017) indicate that general knowledge about the brain is the best “safeguard against believing in neuromyths” (p. 1). German pre-service science teachers specializing in biology receive such knowledge during their university education so that they will later be able to pass it on to their students in their classroom instruction. It can thus be assumed that they develop the theory-based learning beliefs and professional knowledge about neuroscience needed to critically evaluate neuromyths about learning and memory during their university studies. According to Kunter et al. (2013), these two factors, along with motivational orientations and self-regulative skills, are prerequisites for reflective instruction, or in other words, the teaching profession. Kunter et al. (2013) subdivides beliefs into epistemological beliefs and beliefs about learning content and instructional practice. Applying this to the topic of neuroscience and learning, pre-service teachers might have theory-based learning beliefs on the nature of science and teaching and learning. However, they also bring with them beliefs about the definition of learning and learning strategies rooted in their own learning biographies (biography-based learning beliefs). The professional knowledge pre-service science teachers in Germany specializing in biology are expected to acquire during their studies can be subdivided into, inter alia, psychological-pedagogical knowledge (PPK), content knowledge (CK), and pedagogical content knowledge (PCK) (Shulman, 1987; Kunter et al., 2013). Applying this to the topic of neuroscience and learning, pre-service science teachers need to acquire PPK about the psychology of human learning, CK about curricular content related to neuroscience, and PCK about instructional strategies for sustainable learning (Meier et al., 2018). According to Dündar and Gündüz (2016), pre-service science teachers perform significantly better in terms of neuromyths than pre-service teachers in other subjects. As of yet, there are no studies specifically investigating neuromyths about learning and memory or on their prevalence among pre-service science teachers (specializing in biology) depending on their status within teacher education.

As mentioned above, Dekker et al. (2012) and other existing studies on neuromyths interpret the frequently found association between endorsement of neuromyths and scientific concepts as a general tendency to agree with neuroscientific statements among teachers. After more intensive theoretical work on neuromyths, we also see these correlations as rooted in the fact that the test instrument asks in some cases about both the neuromyth and the corresponding kernel of truth as scientific concepts. For example: neuromyth = *“Individuals learn better when they receive information in their preferred learning style (e.g*., *auditory, visual, kinesthetic)“* and scientific concept (kernel of truth) = *“Individual learners show preferences for the mode in which they receive information (e.g*., *visual, auditory, kinesthetic).“* Dekker et al.’s (2012) instrument for scientific concepts, which was applied in many of the previous studies on neuromyths, cannot be seen as an appropriate knowledge test for pre-service science teachers in light of our theoretical perspective on professional knowledge among science teachers. In order to more effectively design professional development offerings, it will be necessary to further clarify the causal relations between misconceptions and aspects of professional competency (beliefs and professional knowledge). The present study is the first to do this.

## Methods

Building upon the aforementioned theoretical work, this study addresses three research questions: (1) How are pre-service science teachers’ misconceptions and scientific concepts about learning and memory associated with their status within teacher education? (2) What misconceptions and scientific concepts do pre-service science teachers have on the topic of learning and memory? (3) How are their misconceptions associated with their beliefs and professional knowledge about the topic of learning and memory?

### Participants

The study was conducted among pre-service science teachers specializing in biology at two German universities (University of Kassel and University of Kiel) as well as several institutes for post-graduate teacher trainees (*Studienseminare*) in the federal state of Hesse (*N* = 550). The total sample consisted of 152 first-year students, 260 advanced students (second year and above), and 138 post-graduate teacher trainees (*Referendare*, who were an average of 9 months into their training, *SD* = 4.47). Respondents were 24.8% male and 75.2% female, and were between 18 and 38 years of age (*M* = 24 years old, *SD* = 3.79). 69.4% of respondents were studying to be teachers at college-preparatory secondary schools (*Gymnasium*), and 30.6% were studying to be teachers in lower-track secondary schools (*Lehramt f
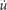
r Haupt- und Realschulen*). Research Question 3 was investigated with a subsample of 79 advanced students, who had the opportunity to participate in a more extensive testing for organizational reasons. Advanced means that they had already completed a human biology course with neuroscience content during their studies. 21.5% of the respondents in this sample were male, while 78.5% were female. The average age was 25 years (*SD* = 2.70) and the respondents were in their eighth semester of studies on average (*SD* = 2.56).

### Procedure

The data was collected in 19 courses in the field of instructional methods for science (biology) education. The post-graduate teacher trainees were recruited and surveyed via their supervisors at the teacher training institutes. Participation took the form of a paper-and-pencil test lasting approximately 15 min. The testing time was expanded to 1 h for a subsample of participants (*n* = 79) in order to apply further instruments (see Materials). In both cases, the project was introduced as a study on the topic of neuroscience and learning; the term “neuromyths” was not used. Participation in the evaluation was voluntary and the students provided informed written consent to use the data for research purposes. They were informed that the goal of the study was to collect information on their current state of knowledge and attitudes toward the topic of neuroscience and learning, and that the anonymity of their data would be ensured via a coding system. They were further notified that they could withdraw from participation at any time without consequences. The authors strictly handled student anonymity and ethical issues.

### Instruments

The test instrument for Research Questions 1 and 2 consisted of 11 items on scientific concepts and 11 items on misconceptions/neuromyths (α = 0.66 and 0.76, respectively^3^). 13 of these 22 items (8 items on scientific concepts and 5 on neuromyths) were taken from Dekker et al. (2012) and translated into German. One item (“Memory is stored in the brain much like as in a computer. That is, each memory goes into a tiny piece of the brain”) was taken from Howard-Jones et al. (2009) and put into more concrete terms: “The brain works like a hard drive; information is stored in specific locations.” To guarantee the fidelity of the translation, the resultant version was back-translated into English by a native speaker and both English versions were compared by a third person. Five neuromyths-items on development (Myth: most receptive to learning before age 3), hemispheric asymmetry (Myth: logic in the left hemisphere, creativity in the right), memory (Myth: Genetically determined number of cells determines learning), learning while you sleep (Myth: You can learn while you sleep, e.g., via audio recordings) and evidence-based learning techniques (i.e., desirable difficulties, Bjork and Bjork, 2011; Myth: Blocked learning is better than interleaved learning) were newly constructed for this study, as they have been widely publicized in German media and learning guides. We followed Macdonald et al. (2017)’s methodological recommendations and replaced the three-option answer format *Correct*/*Incorrect*/*I don’t know* used by Dekker et al. (2012) and other studies of neuromyths with a 4-point Likert scale in order to force respondents to take a position and allow them to specify how sure they were of their answer (4 = *Strongly agree/1 = Strongly disagree*) or how torn (3 = *Somewhat agree/2 = Somewhat disagree*). Because the German versions of some items from Dekker et al. (2012) were refined in terms of content and several new items were created, an English version of the instrument has been provided along with this article (see Appendix). Future studies should note that the German version of the instrument was employed in this study (published in Grospietsch and Mayer, 2018a).

Six instruments on professional knowledge, biography-based learning beliefs, and theory-based learning beliefs on neuroscience and learning were used to answer Research Question 3. Table 2 provides an overview of the instruments and corresponding scales, numbers of items, and reliability coefficients. Example items are provided here; a complete overview of all items can be found in the Supplementary Material. Biography-based learning beliefs were measured via 6-point Likert scales and theory-based learning beliefs via 4-point Likert scales. Professional knowledge was measured via three self-constructed knowledge tests. CK about curricular content in neuroscience was measured via six multiple-choice items with four distractors each. PCK about instructional strategies for sustainable learning (including how to deal with students’ misconceptions about the structure and function of the brain) was measured via 12 open-ended and closed-ended questions, and PPK about the psychology of human learning via 17 open-ended and closed-ended items. The differences in test construction are rooted in the project’s research focus. The interrater reliability for all open-ended items was found to be Cohen’s κ = 0.91 (*p* < 0.001). This indicates almost perfect agreement (Landis and Koch, 1977).

**Table 2:** Overview of the instruments for learning beliefs and professional knowledge.

Instrument			Scales (Number of items)	Example Item (translated from German)	α^∗4^
Learning Beliefs	biography-based	Definition of learning adapted from Drechsel (2001)	• as reproduction (7) • as transformation (7)	*I connect learning in university with:* •*grasping something* •*committing something to memory*	0.58 0.83
		Learning strategies adapted from Ruffo (2010)	• Use of cognitive learning strategies (7) • Use of metacognitive learning strategies (7)	*How well do you relate to the following statements?* •*In order to organize the material, I often make outlines, tables and sketches.* •*I consider while working whether my approach up to now makes sense*	0.70 0.72
	theory-based	Beliefs about teaching and learning Gimbel et al. (2018) adapted from Seidel and Meyer (2003)	• transmissive (7) • constructivist (7)	*How do students learn science (biology)?* •*Teachers should always give detailed instructions as to how biology experiments should be done.* •*One should enable students to independently find solutions to biology tasks before the teacher demonstrates how to solve them.*	0.76 0.80
		Nature of science Gimbel et al. (2018) adapted from Urhahne et al. (2008), Riese (2009)	Nature of science (28)	*What do you think about science (biology)?* •*Biological knowledge isn‘t definitively provable and can change over time.*	0.95
Professional Knowledge		CK Newly constructed	Curricular content on neuroscience (6)	*Which part of the brain is responsible for motor skills?* ○ *The cerebellum* ○ *The cerebrum* ○ *The corpus callosum* ○ *The forebrain*	0.60
		PCK Newly constructed	Instructional strategies for sustainable learning (12)	*Name three school experiments on the topic of learning.*	0.72
		PPK Newly constructed	Psychology of human learning (17)	*Which of the following are components of working memory?* ○ *Central executive* ○ *Semantic memory* ○ *Iconic storage* ○ *Phonological loop*	0.80

*^∗^The Cronbach’s alpha values generally refer to a sample of *n* = 79 students; for the knowledge tests, they refer to a subsample (*n =* 40) who filled out the instrument as a post-test after participating in a learning environment on the topic of neuroscience and learning.*

In addition, information on sociodemographic data (age, gender, field of study, years of study/training, enrolled in university courses on neuroscience and learning) were collected for all participants. Except for years of study/training and enrollment in a human biology course, these demographic data were requested for descriptive purposes only and were not explored further in the subsequent analyses.

### Data Analysis

We used multifactorial analyses of variance to test whether first-year students, advanced students, and post-graduate teacher trainees differed in their endorsement of scientific concepts and neuromyths (Research Question 1). These three groups were considered to be in different stages of teacher education due to differences in educational content: Group 1 was enrolled in introductory courses in instructional methods in science (biology) education, disciplinary content, and education science; Group 2 was enrolled in or had completed more advanced education science and subject-specific instructional methods courses on teaching and learning (in science) as well as modules that covered neuroscience (human biology and/or animal physiology); and Group 3 was in the process of completing a practical training phase after university graduation. One-way analyses of variance and Bonferroni-adjusted *post hoc* analyses were used to determine the extent to which the groups differed in their endorsement of individual neuromyths. One-way analyses of variance with Welch corrections and Games-Howell *post hoc* analyses were applied in the case of heterogeneous variance. To this end and to answer Research Question 2, the 4-point Likert scale was recoded into a dummy format (*agree/disagree*) for better comparability with the previously cited studies. First, the percentage of respondents who agreed with each item (both neuromyths and scientific concepts) was calculated. Then, the neuromyths/scientific concepts were grouped by content into different neuroscientific topics (categories) on the basis of theory. If a category had more than one item, the mean of the percentages was taken. Correlation analyses (Pearson) were conducted to determine the associations between endorsement of neuromyths and beliefs about learning and memory (biography-based learning beliefs about the definition of learning at university and use of learning strategies as well as theory-based beliefs about the nature of science and teaching and learning), professional knowledge (CK about curricular content related to neuroscience, PCK about instructional strategies for sustainable learning, and PPK about the psychology of human learning). The significance level for all analyses was *p* ≤ 0.05.

## Results

### Scientific Concepts and Misconceptions by Status Within Teacher Education

**FIGURE 1:**
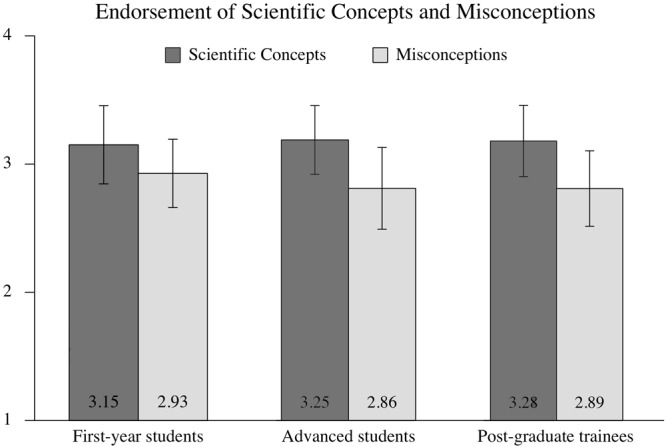
Group comparison on endorsement of scientific concepts (left) and misconceptions (right) (mean and standard deviations are presented; 4 = strongly agree, 3 = somewhat agree, 2 = somewhat disagree, 1 = strongly disagree).

A multifactorial analysis of variance revealed a significant main effect for conception type (misconceptions vs. scientific concepts: *F*(1,1076) = 311.70, *p* ≤ 0.001, $\eta_{p}^{2}$ = 0.225) but not for stage of teacher education [first-year students, advanced students, and post-graduate teacher trainees: *F*(2,1076) = 1.95, *p* = 0.143, $\eta_{p}^{2}$ = 0.004]. There was a statistically significant interaction between stage of teacher education and conception type: *F*(2,1076) = 8.13, *p* ≤ 0.001, $\eta_{p}^{2}$ = 0.015. The mean levels in Figure 1 show that respondents in different stages of teacher education differed from one another in their endorsement of scientific concepts (left) but not in their endorsement of neuromyths (right).

Turning to the percentage agreeing with individual neuromyths (dichotomous answer format), one-way analyses of variance only uncovered differences between the three groups of subjects with respect to the neuromyths on critical periods of childhood development [Welch’s *F*(2,297) = 11.84, *p* ≤ 0.001], blocked learning is better than interleaved [Welch’s *F*(2,305) = 4.80, *p* = 0.009], the existence of learning styles [Welch’s *F*(2,325) = 3.58, *p* = 0.029] and a genetically determined number of cells determines learning [*F*(2,533) = 24.29, *p* ≤ 0.001]. Games-Howell *post hoc* analyses revealed significantly greater agreement with the myth of critical periods of childhood development among advanced students compared to first-year-students and post-graduate trainees (68 vs. 56%, *p* = 0.049, 0.12, 95%-CI[0.00, 0.24] and 43%, *p* ≤ 0.001, 0.25, 95%-CI[0.13, 0.37]). The neuromyth that blocked learning is better than interleaved was more frequently rejected by advanced students and post-graduate trainees than by first-year students (49% *p* = 0.021, -0.14, 95%-CI[-0.27, -0.02] and 46% *p* = 0.017, -0.16, 95%-CI[-0.3, -0.02] vs. 62%). The neuromyth on the existence of learning styles was significantly less frequently endorsed by advanced students than by first-year students (90 vs. 97%, *p* = 0.023, -0.06, 95%-CI[-0.12, -0.01]). A Bonferroni-adjusted *post hoc* analysis revealed that the neuromyth that a person’s genetically determined number of cells forms an upper limit for learning success was actually endorsed significantly (*p* ≤ 0.001) more by post-graduate trainees (64 vs. 33% of first-year students 0.32, 95%-CI[0.18, 0.45], and 31% of advanced students, 0.33, 95%-CI[0.21, 0.45]).

### Endorsement of Misconceptions and Scientific Concepts

**FIGURE 2:**
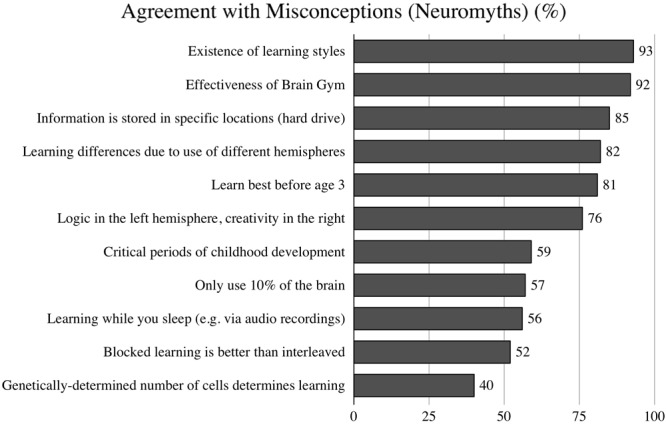
Agreement with misconceptions (neuromyths) among all participants.

Pre-service science teachers’ neuroscience literacy was to a large extent rooted in neuromyths (Figure 2). 10 of 11 misconceptions on the topic of learning and memory were endorsed by more than half of respondents. The existence of learning styles, the effectiveness of Brain Gym, and the notion that information is stored in specific locations (hard drive) were endorsed most frequently (with 93, 92, and 85% of respondents agreeing with these items, respectively). The only neuromyth to be endorsed by fewer than half of the pre-service teachers in the sample was the notion that a person’s genetically determined number of cells determines learning success (with 40% of respondents agreeing).

Neuromyths were sometimes endorsed even when respondents had thematically similar scientific concepts (Table 3). This was seen in the categories of development, memory, learning techniques, brain activity, and sensory modalities. On the other hand, high levels of agreement with neuromyths were found in categories in which fewer respondents had thematically similar scientific concepts. This was the case for the categories of neuroplasticity and hemispheric asymmetry.

**Table 3:** Comparing endorsement of scientific concepts and misconceptions.

Scientific concept	Agreement (%)	Misconception	Agreement (%)
**Development**			
Certain phases of childhood are more sensitive for learning (e.g., for language acquisition)	98	e.g., most receptive to learning before age 3 (*two items*)	50
**Memory**			
E.g., learning is based on changes in neural connections (*two items*)^∗^	95	e.g., the brain works like a hard drive; information is stored in specific locations (*two items*)	63
**Learning techniques**			
E.g., testing effect (desirable difficulties) (*two items*)	94	blocked learning is better than interleaved	52
**Brain activity**			
E.g., the brain is active 24 h a day (*two items*)	93	learning while you sleep over the acoustic channel	56
**Sensory modalities**			
Visual, auditory, etc. reception of information	92	Existence of learning styles	93
**Neuroplasticity**			
When a brain region is damaged, other parts of the brain can take up its function	60	Only use 10% of brain	57
**Hemispheric asymmetry**			
The hemispheres work together	40	E.g., Brain Gym better links the two hemispheres (*three items*)	83

*^∗^If a category has more than one item, the percentages were added together.*

### Correlations With Beliefs and Professional Knowledge

As can be seen in Table 4, the advanced students for whom individual aspects of professional competency were investigated tended toward agreement with respect to biography- and theory-based learning beliefs. Transmissive beliefs were endorsed to a lesser extent than constructivist beliefs. The latter received the highest average agreement alongside nature of science beliefs. On the knowledge tests, the advanced students were able to correctly answer about 50% of the CK, 20% of the PCK, and 30% of the PPK questions. The standard deviations here varied more widely than they did for beliefs.

**Table 4:** Correlations of misconceptions with learning beliefs and professional knowledge.

Aspects of professional competency			*M* (*SD*) /Max score	Correlation with misconceptions		
				*r*	*p*	*n*
Learning Beliefs	biography-based	Definition of learning				79
		as reproduction	3.92 (0.57)/6	0.158	0.164	
		as transformation	3.69 (0.86)/6	0.071	0.532	
		Learning strategies				79
		cognitive	3.39 (0.79)/6	0.113	0.321	
		metacognitive	3.57 (0.69)/6	0.027	0.811	
	theory-based	Beliefs about teaching and learning				75
		transmissive	2.45 (0.49)/4	0.139	0.236	
		constructivist	3.50 (0.42)/4	0.313^∗∗^	0.006
		Nature of science	3.49 (0.34)/4	–0.090	0.444	75
Professional Knowledge						75
		CK about curricular content related to neuroscience	3.00 (1.41)/6	0.091	0.437	
		PCK about instructional strategies for sustainable learning	4.83 (2.46)/24	–0.062	0.595	
		PPK about psychology of human learning	9.85 (3.24)/34	0.062	0.600	

Correlational analyses revealed only a small positive correlation between neuromyths und constructivist beliefs about teaching and learning (*r* = 0.313, *p* = 0.006). This correlation within the area of theory-based learning beliefs means that pre-service science teachers who endorse misconceptions also exhibit a constructivist view of teaching and learning and think of learning as an active, self-directed, constructive process in which knowledge cannot simply be transferred to the learner (Staub and Stern, 2002). No correlations were found between misconceptions and the other theory-based learning beliefs (transmissive and nature of science beliefs) or the three areas of professional knowledge on neuroscience and learning (CK, PCK, PPK). Nor were there any significant correlations between misconceptions and biography-based learning beliefs related to respondents’ subjective definition of learning or inventory of learning strategies (Table 4).

## Discussion

### Pre-service Science Teachers’ Scientific Concepts

The results of our study demonstrate that pre-service science teachers’ scientific concepts on learning and memory increase over the course of their training. This finding is in accordance with expectations, because German science teachers specializing in biology complete modules containing neuroscientific content (human biology and animal physiology) during their university studies. It should be noted that average endorsement of scientific concepts did not increase dramatically and was quite high even among first-year students. Based on these findings, one could conclude that many pre-service biology teachers have already acquired scientific concepts during school (e.g., in advanced high school biology courses) and bring them with them to university. However, from a critical perspective, it should be noted that our instrument did not allow us to measure what the students actually know and when they simply took a position despite a lack of knowledge (intuiting/guessing). This problem was strengthened by our use of a Likert scale, which was however recommended by Macdonald et al. (2017). Despite differences to Dekker et al.’s (2012) instrument, our survey was able to confirm their finding that there is a general tendency to agree with neuroscientific statements. Our results indicate that this tendency persists despite academic and practical training.

Our results further indicate that the pre-service science teachers in our study have the weakest scientific concepts with respect to neuroplasticity and hemispheric asymmetry (60 and 40%). This could be because these topics tend to be covered only marginally or as an aside in neuroscience courses and textbooks. From a critical perspective, it should be noted that these values are location-specific and could be different at other German universities. There is currently no curriculum stipulating which scientific concepts must be covered as part of science teachers’ training in the fundamentals of neuroscience. One item from each of the two aforementioned categories adopted from Dekker et al. (2012) have also been employed in other studies of neuromyths. Putting aside the differences in answer format (our 4-point Likert Scale vs. *correct/incorrect/I don’t know*) and the slightly more concrete items in our translation, comparing our results to previous studies indicates that German pre-service science teachers have stronger scientific concepts than Turkish and British pre-service teachers with respect to the items “*When one brain region is damaged due to injury, other parts of the brain can take up its function*” and “*The left and right hemispheres of the brain always work together in processing information*” (Dündar and Gündüz, 2016: 20 and 15% correct answers, Papadatou-Pastou et al., 2017: 12 and 14% correct answers, although around 30% of respondents in both studies selected *I don’t know*). The pre-service science teachers in this study endorsed the topics of development, memory, learning techniques, brain activity, and sensory modalities at very high rates (98-92%). The values of all matching items were higher than in the study by Papadatou-Pastou et al. (2017), although the instruments’ differences in language and answer format must be taken into account. The presented tendencies concerning overlapping items indicate that pre-service science teachers seem to have stronger scientific concepts related to neuroscience than other pre-service teachers. This should be tested in a study employing the same instruments for both groups of participants.

### Pre-service Science Teachers’ Misconceptions (Neuromyths)

Given that this study’s quasi-longitudinal design found no differences in endorsement of neuromyths between *first-year students, advanced students*, and *post-graduate trainees*, teacher education does not seem to be able to successfully professionalize students’ misconceptions about learning and memory. Only two neuromyths about learning and memory (critical periods and blocked learning) were endorsed less by post-graduate teacher trainees, who have already completed their university training in neuroscience and learning, than by students still in university. In fact, the neuromyth that a person’s genetically determined number of cells forms an upper limit for learning success was endorsed more frequently among the group of post-graduate trainees. This increase is alarming, as belief in this type of myth bestows upon or denies learners a pre-determined, non-malleable aptitude for learning. This could have consequences for teachers’ interactions with students and thus also for students’ self-efficacy beliefs. Whether and to what extent these individual neuromyths find their way into pre-service science teachers’ later pedagogical practice is not clear on the basis of our study. We join Horvath et al. (2018) in arguing that future studies must test the extent to which the endorsement of neuromyths influences teachers’ effectiveness. However, other studies show that teachers’ beliefs and attitudes guide their actions (Wahl, 1979). We assume that university education represents the most significant opportunity for German science teachers to acquire neuroscientific knowledge in a guided way. In light of the previously cited studies of pre-service and in-service teachers in all school subjects (e.g., Dekker et al., 2012) revealing comparatively high levels of endorsement of neuromyths, we do not assume that in-service science teachers endorse misconceptions to a lesser extent than the pre-service teachers in our study. It might even be the case that committed efforts among in-service teachers to optimally guide students’ learning lead to greater use of practical approaches based on neuromyths, such as Brain Gym or learning styles. In this way, neuromyths might be even more widespread among German in-service science teachers than pre-service teachers. However, further comparative and longitudinal studies are necessary to investigate these hypotheses. In any event, teacher education as it currently exists in Germany does not seem sufficient to dismantle misconceptions about learning and memory or replace them with scientific concepts. New, more effective professional development opportunities and learning programs must be created.

The results presented in this study confirm previous findings that it is not just in-service teachers who believe in neuromyths – a large share of pre-service teachers endorse them as well (Howard-Jones et al., 2009; Fuentes and Risso, 2015; Dündar and Gündüz, 2016; Canbulat and Kiriktas, 2017; Düvel et al., 2017; Kim and Sankey, 2017; Papadatou-Pastou et al., 2017; Im et al., 2018). Out of a total of 11 misconceptions (neuromyths) about learning and memory, the existence of learning styles (93%), the effectiveness of Brain Gym (92%), and the assumption that information is stored in specific locations (hard drive) (85%) were endorsed most frequently. Comparing our results to those of other studies (despite the difference in answer format and the slightly more concrete items in our translation), these myths were also quite frequently endorsed by pre-service teachers in Turkish (Dündar and Gündüz, 2016: 97, 67, and 79%) and British studies (Howard-Jones et al., 2009: 82, 62, und 36%, Papadatou-Pastou et al., 2017: 94, 37% und -%)^4^. There thus appears to be a core group of neuromyths whose prevalence is independent of culture.

Furthermore, German pre-service teachers believe more frequently than Turkish (Dündar and Gündüz, 2016) or British pre-service teachers (Howard-Jones et al., 2009; Papadatou-Pastou et al., 2017) in the neuromyths of only using 10% of our brain (57% compared to 42, 52 and 47%) and critical periods of childhood development (item = *If the brain is not sufficiently supported in early childhood, learning problems that can no longer be remediated by education can occur*: 59% compared to 59, 9, and 24%)^5^. Comparing our results to those of Papadatou-Pastou et al. (2017), it appears that German pre-service teachers believe more strongly in the myths of learning differences due to the use of different hemispheres (82% vs. 55%, although *I don’t know* was selected quite frequently in Papadatou-Pastou et al., 2017)^5^ and learning while you sleep (56 vs. 38%)^5^. This points to cultural differences in levels of agreement with individual neuromyths among pre-service teachers, just as among in-service teachers. Moreover, it indicates that the characterization of neuromyths as ‘inadequate scientific concepts’ is insufficient. Instead, these cultural differences suggest that neuromyths to a large degree feed off of a socio-cultural discourse that finds its specific expression – cultural differences included – in these neuromyths. Thus, these misconceptions might be better described as scientific myths (cf. Papadatou-Pastou et al., 2017). Empirical inquiries and interventions should take socio-cultural discourses about teaching and learning into consideration as the relevant context of neuromyths. This study also found evidence for endorsement of the myth that blocked learning is more effective than interleaved learning (i.e., desirable difficulties, Bjork and Bjork, 2011) for the first time. Thus, there is a need for action in teacher education with respect to the aforementioned neuromyths, particularly in light of the importance of a professional understanding of learning and memory for instructional content and instructional methods in science.

### Relations Between Neuromyths and Aspects of Professional Competency

Turning to the aspects of professional competency, firstly, our results with respect to theory-based learning beliefs were in accordance with expectations. Our hypothesis that constructivist and nature of science beliefs would be stronger among advanced students were confirmed. In accordance with the existing research literature (e.g., Brauer et al., 2014), transmissive beliefs about teaching and learning were in turn less strong among these students. Thus, for these students, learning is more of an active than a passive process in which knowledge can be generated independently. This can be interpreted together with professional beliefs on the origins of knowledge in biology (nature of science) as evidence in favor of a professional understanding of learning and memory. The students’ biography-based learning beliefs can also be positively interpreted in light of the rather strong deployment of learning strategies and the definition of learning as transformation (professional definition of learning). However, the existing research literature (Drechsel, 2001) also indicates that advanced students maintain less professional definitions of learning (learning as reproduction). In fact, in our study, these were even more widespread than the professional ones. Whether and to what extent these beliefs influence the students’ later actions in schools remains an open question. Our results further indicate that students have some knowledge of PCK about instructional strategies for sustainable learning and PPK about the psychology of human learning, but not a great deal. We see this as primarily rooted in our methodological decision to select advanced students who had completed human biology courses with neuroscientific content. We have no information on the extent to which this sample is also at an advanced level with respect to educational science, psychology, and instructional methods course. However, the CK about curricular content in neuroscience we selected for was present to a stronger extent, although this could also be rooted in the closed answer format. The larger standard deviations for the knowledge results point to differences in the students‘ performance.

Our results show that all of the aspects of professional competency we investigated were present among the students. However, there were few correlations with endorsement of neuromyths. Building upon studies that call for more neuroscience in teacher education (e.g., Papadatou-Pastou et al., 2017), we expected negative correlations with students’ professional knowledge, which would mean that students with greater knowledge endorse neuromyths to a lesser extent. However, no such correlations were found for CK about curricular content related to neuroscience, PCK about instructional strategies for sustainable learning or PPK about the psychology of human learning. For PCK and PPK, this might be rooted in the students’ low levels of knowledge or methodologically in the difficulty level of the tests, which was too high. We reject this possibility with respect to CK about curricular content related to neuroscience, as the students had more knowledge here. Our results indicate that neuromyths are independent of CK. However, it must be emphasized that our instrument only asked about curricular content in neuroscience with respect to the topics of brain structure, memory, and long-term potentiation commonly found in school textbooks. General knowledge of neuroscience and the latest research findings were not considered in this study and could still be a predictor of endorsement of neuromyths.

In addition, the results of our study revealed no correlations with respect to a person’s learning at university (definition of learning and learning strategies), despite our theoretical assumptions indicating that less professional biography-based learning beliefs could facilitate the endorsement of neuromyths. Perhaps our scale on learning as reproduction, with α = 0.58, could not measure the theoretical construct sufficiently and accurately enough. Future studies could also ask about concrete learning experiences that could promote neuromyths (e.g., if they conducted learning style tests during their school years). However, this study found a small positive correlation between misconceptions and constructivist beliefs about teaching and learning despite the fact that some neuromyths (e.g., that the brain works like a hard drive) are not theoretically compatible with such beliefs. People with constructivist views of teaching and learning actually view learning as an active, self-directed, constructive process in which knowledge cannot simply be transferred to the learner (Staub and Stern, 2002). Consequently, neuromyths seem to be integrated into the semantic network of theory-based learning beliefs despite their scientific inconsistencies, which can make them more difficult to change. The co-existence or even synthesis of misconceptions and theory-based beliefs are well-established in theories of misconceptions and conceptual change (Vosniadou, 2013). Studies by Petitto and Dunbar (2004) describe how university students can stubbornly hold onto their original concepts despite empirical demonstrations and theoretical explanations. Newton and Miah (2017) demonstrated this specifically for the neuromyth on the existence of learning styles. In addition, the authors warn of a “backfire effect,” a phenomenon in which attempts to address myths and misunderstandings can lead to a strengthening of beliefs in these myths. Thus, the results of this study indicate that interventions against the endorsement in neuromyths must begin deep in participants’ belief systems.

### Implications for Intervention Approaches With Respect to Neuroscience Literacy

Overall, our results for pre-service science teachers show that Papadatou-Pastou et al.’s (2017) call to integrate neuroscientific content into teacher education is not in itself sufficient to limit the spread of neuromyths. Even these students, who are taught such content in their courses, must be trained to become critical consumers of neuroscientific research findings (a kind of preventative focus; Macdonald et al., 2017).

In line with the previously cited neuromyth studies, our findings confirm the need for action with respect to neuromyths. Few intervention approaches have been proposed. Papadatou-Pastou et al. (2017) stresses the importance of developing an understanding of how neuroscience research is conducted and presented (e.g., imaging techniques using differential images). This might not be occurring to a sufficient extent in German pre-service science teacher education, which is primarily set up to enhance professional knowledge. Neuroscience will always be in a continuous state of development and progress. Pre-service science teachers should be put in a position in which they are able to follow the latest developments by effectively reading and critically evaluating the information they obtain from various sources.

According to Guzzetti et al. (1993) and Kowalski and Taylor (2009, 2011), one of the most effective evidence-based methods of addressing scientific myths consists of directly refuting misunderstandings. Grospietsch and Mayer (2018b) have confirmed this for neuromyths. Neuromyths arise and persist from an entire line of argumentation consisting of misinterpretations and exaggerations that can only be refuted with a multitude of neuroscientific facts (examples provided in Table 1 of this study). This speaks in favor of examining each neuromyth individually, determining its “kernel of truth,” uncovering its argumentation structure, and comparing it to the corresponding scientific concepts. Only by investigating each neuromyth individually and in more detail than previously will we be able to determine why neuromyths have been spreading and develop appropriate interventions to stop them. This requires strong cooperation between education, neuroscience, and cognitive psychology. MacNabb et al. (2006) demonstrate the positive effects of such cooperation. Materials and courses counteracting the false transfer of scientific concepts to classroom teaching and learning need to be jointly developed. A web of exchange between the field of education and neuroscientists is required to put neuromyths into a more scientifically accurate light. Neuroscientists’ assistance is particularly necessary when it comes to incorporating the latest research.

Several neuromyths found among pre-service science teachers in this study have also been frequently demonstrated in international studies of in-service teachers (cf. e.g., Dekker et al., 2012; Ferrero et al., 2016). Given that even first-year students exhibit beliefs in neuromyths, it is likely that pre-service teachers encounter these misconceptions even before beginning their university studies, i.e., during school. It is well-known that neuromyths such as the theory of learning styles and Brain Gym exercises are found in a large number of learning guides and educational programs (Pasquinelli, 2012). Students can also encounter neuromyths through their teachers. Studies by Schletter and Bayrhuber (1998) provide empirical indications that misconceptions (e.g., that the brain works like a hard drive) are present among school students. To the best of our knowledge, there are not yet any studies systematically investigating the spread of neuromyths about learning and memory among school students. What we do know is that the misconceptions that develop over a person’s school years are difficult to change via formal education at university (Pajares, 1992). Starting intervention during students’ school years or at the beginning of university education seems advantageous.

### Recommendations for Future Studies

As previously discussed, the results of our study show that pre-service science teachers have weak scientific concepts on neuroplasticity and hemispheric asymmetry. Our results further show that the low levels of scientific concepts for these topics were accompanied by high levels of endorsement of thematically similar neuromyths. Based on our findings, one might conclude that these topics need to be more strongly integrated into teacher education and associated neuroscience teaching materials. Even though pre-service science teachers endorsed scientifically accurate statements (scientific concepts) about the topics of development, memory, learning techniques, brain activity, and sensory modalities, the thematically similar neuromyth items were widely endorsed as well (50–93%). In these cases, we concur with Dekker et al. (2012) and follow-up studies by other authors that there is a lack of ability to differentiate scientific concepts from misconceptions. Endorsement of misconceptions (neuromyths) was not lower for topics in which there was less knowledge of scientific concepts (93% for learning styles despite high endorsement of scientific concepts; 92% for Brain Gym amid low endorsement of scientific concepts). Consequently, the results obtained with our survey suggest two different causes for the emergence of neuromyths: a lack of scientific concepts, but also false transfer of accurate scientific concepts to teaching and learning. Future studies should further clarify these different explanations for the emergence of neuromyths. In doing so, it would be advantageous to not only contrast thematically similar items on scientific concepts and neuromyths, as we did in the questionnaire for this study, but rather to consistently identify and inquire about the kernel of truth and unique argumentation errors for each neuromyth (see Table 1). Given the current state of theoretical work on neuromyths, we were only able to do this for a few categories (e.g., sensory modalities). Much more theoretical work on neuromyths needs to be completed with respect to this issue. With regard to intervention approaches, future studies must also test whether the theoretical argumentation in favor of neuromyths previously described actually conform to those held by pre-service and in-service teachers. Thus, we see a need for more intensive empirical and theoretical research on neuromyths.

In this study, we were able to show that pre-service science teachers endorse a variety number of neuromyths. We did not collect data on the sources of the students’ neuroscientific information or their perceptions of the origin of their misconceptions on the topic of learning and memory. Biography-based learning experiences in the everyday world, independent learning by reading scientific/leisure magazines, or even the structure of university trainings could be potential sources of neuromyths. Following Yeung et al. (2018), the readability of neuroimaging articles and their abstracts could be particularly problematic, especially for first-year students. All of these aspects should be more thoroughly investigated in future studies. In our opinion, qualitative studies in which students are asked to describe their arguments in favor of neuromyths seem more worthwhile than surveys of various sources of information.

### Summary and Outlook

In summary, our results indicate that neuromyths can exist in parallel to scientific concepts, professional knowledge and beliefs about neuroscience and learning and are resistant to conventional German teacher education, which promotes many aspects of professional competency on this topic. From the perspective of conceptual change theory, neuromyths thus exhibit characteristic traits of misconceptions that cannot simply be counteracted with increased neuroscientific knowledge. Both neuroscientific knowledge and didactic interventions will be required to effectively and sustainably banish neuromyths from the education system. For this reason, we call for stronger cooperation between neuroscientists and didactics experts. Creating links between education/didactics on the one hand and neuroscience and cognitive psychology on the other seems to be essential for confronting neuromyths’ lines of argumentation with scientific knowledge as well as improving science teachers’ neuroscience literacy. According to the Organization for Economic Co-operation and Development [OECD] (2008), each discipline has its own specific methods and language, which makes it particularly difficult for experts in one area to apply knowledge from the other. Joint publications and training programs for pre-service and in-service teachers on the cognitive errors involved in neuromyths would be an important first step toward eliminating ‘language barriers’ (Pickering and Howard-Jones, 2007) and closing the gap between neuroscience and the practice of education (Edelenbosch et al., 2015), at least with respect to neuromyths. Teachers train the neuroscientists of tomorrow. It is therefore important to take teachers seriously, to investigate how neuroscience can help them better understand learning, and to invest in their ability to optimally use neuroscience in their practice.

On the basis of this study’s results, the University of Kassel has developed a learning environment in accordance with the conceptual change model through interdisciplinary cooperation. This learning environment gives students reasons and opportunities to more closely interlink their professional knowledge in neuroscience, cognitive psychology, and instructional methods in science and to critically question incomplete or incorrect misconceptions and beliefs about learning and memory. An accompanying study demonstrated positive results with respect to pre-service science teachers’ neuroscience literacy (Grospietsch and Mayer, 2018b).

## Data Availability Statement

The raw data supporting the conclusions of this manuscript are available on Open Science Framework. The link will be provided by the authors upon request.

## Ethics Statement

No ethics approval was required for the reported study as per the guidelines of the University of Kassel or national guidelines. We conducted the study in line with the recommendations of the University of Kassel’s ethics committee. All participants gave written informed consent in accordance with the Helsinki Declaration.

## Author Contributions

JM was responsible for the project administration and funding acquisition. Both authors developed the study concept and the methodology. FG developed the study materials, conducted the data collection, and analyzed the data. This article is part of the Ph.D. thesis of FG supervised by JM. FG drafted the manuscript. JM provided critical revisions. Both authors approved the final version of the manuscript for submission.

## Conflict of Interest Statement

The authors declare that the research was conducted in the absence of any commercial or financial relationships that could be construed as a potential conflict of interest.

## Footnotes

1 The term “misconceptions” was selected in this article in order to express that the theoretical assumptions, lines of argumentation, and conclusions drawn from neuromyths violate the rules of scientific thought and argumentation. Thus, we consider misconceptions to be inadequate scientific concepts that stand in clear contrast to scientific concepts.

2 Howard-Jones (2014) provides a comparative overview of some of these studies.

3 The Cronbach’s alpha values for these scales refer to a subsample of advanced students (*n =* 75) who filled out the questionnaire as a post-test after participating in a learning environment on the topic of neuroscience and learning.

4 Agreement with the neuromyths on the brain as a hard drive and Brain Gym was not quite as high in the English studies because the answer option *I don’t know* was selected very often.

5 The items were formulated slightly differently in the cited studies.

## Appendix

Translation of the German instrument on conceptions of learning and memory.

**Table:**

Title of the instrument	Conceptions on learning and memory (all items positively formulated = scientifically accurate)	
Introductory text	Questionnaire on Learning and the Brain. The following statements concern learning and the brain. Please read through the following statements carefully, marking your level of agreement with each. Please answer honestly and select only one answer option for each statement. Make sure not to skip any statements.	

**Scale: Scientific concepts scale**		***r*_it_**

To what extent do you agree with the following statements?		
MEM	Learning occurs through modification of the brains’ neural connections.^1^	0.48
MEM	The forging of new connections in the brain can continue into old age.^2^	0.30
HEM	The left and right hemispheres of the brain always work together in processing information.^2^	0.33
BA	Our brains are active 24 h a day.^2^	0.29
BA	Processes to consolidate what we have learned occur during sleep.	0.40
DEV	There are sensitive periods in childhood when it’s easier to learn things.^1^	0.52
SEN	Individual learners show preferences for the mode in which they receive information (e.g., visual, auditory, kinesthetic).^1^	0.22
LT	Learners’ cognitive abilities can improve with intensive training.	0.32
LT	Learning material can be remembered longer when it is actively worked through rather than read.	0.32
NEU	When one brain region is damaged due to injury, other parts of the brain can take up its function.^2^	0.39
GEN	Male brains are bigger than female brains.^2^	0.22
	Cronbach’s alpha (α) 0.66	

**Scale: Misconceptions scale (neuromyths)** (all items positively formulated = neuromyths)		***r*_it_**

To what extent do you agree with the following statements?			
MEM	The brain works like a hard drive. Information is stored at specific locations.^3^	0.58
MEM	Our genetically determined number of brain cells determines the maximum level at which we can learn.^4^	0.32
HEM	The right brain hemisphere is more involved in creative thought processes, and the left in logical thought processes.	0.31
HEM	Every person uses the right and left hemispheres to a different extent. This can explain differences amongst learners.^2^	0.45
HEM	Short bouts of co-ordination exercises can improve the interaction between the left and right hemispheres.^2^	0.23
BA	It is possible to learn while we sleep via the acoustic channel (e.g., audio recordings of vocabulary lists).	0.51
DEV	If the brain is not sufficiently supported in early childhood, learning problems that can no longer be remediated by education can occur.^2^	0.43
DEV	Learners are most receptive to learning processes from birth to the third year of life.^4^	0.46
SEN	Individuals learn better when they receive information in their preferred learning style (e.g., auditory, visual, kinesthetic).^1^	0.50
LT	Learners perform better when they are able to study different topics systematically one-by-one rather than intermingled with one another.	0.34
NEU	We only use 10% of our brain.^1^	0.43
	Cronbach’s alpha (α) 0.76	

*N =* 76; answer format = 4-point Likert scale (1 – disagree, 2 – somewhat disagree, 3 – somewhat agree, 4 – agree); ^*1*^according to Dekker et al. (2012).; ^*2*^concretized on the basis of items from Dekker et al. (2012); ^*3*^based on Howard-Jones et al. (2009), concretized in accordance with Schletter and Bayrhuber (1998); ^*4*^developed on the basis of Bellert and Graham (2013); MEM = memory; HEM = hemispheric asymmetry; BA = brain activity; DEV = development; SEN = sensory modalities; LT = learning techniques; NEU = neuroplasticity; GEN = gender differences; *r*_*it*_, discrimination parameters, listed here for the German translations of the items.
