# Supplementary material for: Pre-service Science Teachers’ Neuroscience Literacy: Neuromyths and a Professional Understanding of Learning and Memory
Source: Front Hum Neurosci. 2019 Feb 14;13:20. doi: 10.3389/fnhum.2019.00020 (PMC6413703; doi:10.3389/fnhum.2019.00020)
Supplement: Supplementary file 1 [file Data_Sheet_1.pdf]

Instrumente zur Messung von Professionswissen und Überzeugungen zum Thema Lernen und Gedächtnis [Instruments for measuring professional knowledge and beliefs on the topic of learning and memory]

Zitationsvorschlag: Grospietsch, F. & Mayer, J. (2018). Instrumente zur Messung von Professionswissen und Überzeugungen zum Thema Lernen und Gedächtnis. Supplementary Material zu Pre-service Science Teachers' Neuroscience Literacy: Neuromyths and a Professional Understanding of Learning and Memory. *Front. Hum. Neurosci. 13:*20. doi: 10.3389/fnhum.2019.00020

*** Kontakt:**
Finja Grospietsch
finja.grospietsch@uni-kassel.de

Instrument zum Fachwissen (6 Items)

FWBF1

Kreuzen Sie richtig an (1 Antwort):

Welcher Teil des Gehirns ist für die Motorik zuständig?

- ◯ das Kleinhirn
- ◯ das Großhirn
- ◯ das Zwischenhirn
- ◯ das Vorderhirn

FWBF2

Kreuzen Sie richtig an (1 Antwort):

Wo im Gehirn werden lebenserhaltende Basisfunktionen wie Herzschlag/Atmung gesteuert?

- ◯ in der Insula
- ◯ im Hirnstamm
- ◯ im limbischen System
- ◯ im präfrontalen Kortex

FWGW3

Kreuzen Sie richtig an (1 Antwort):

Wie viele Einheiten kann das Arbeitsgedächtnis verarbeiten?

- ◯ circa 7 Informationseinheiten
- ◯ maximal 10 Informationseinheiten
- ◯ maximal 4 Informationseinheiten
- ◯ circa 21 Informationseinheiten

FWGW5

Kreuzen Sie richtig an (1 Antwort):

Was ist keine korrekte Aussage über Wissensarten?

◯ Deklarative Gedächtnisinhalte werden als explizit, perzeptive und prozedurale Inhalte als implizit bezeichnet.

◯ Begriffe und Assoziationen zwischen ihnen sind Teil des semantischen Gedächtnisses und können durch semantische Netzwerke dargestellt werden.

◯ Wort-, Objekt- und Raumformen gelten als Inhalte des perzeptiven Gedächtnisses.

◯ Episodische Inhalte werden im prozeduralen Gedächtnis gespeichert.

FWGW6

Kreuzen Sie richtig an (1 Antwort):

Was fällt unter die Gedächtnisart Priming?

◯ anhand weniger Töne eine Melodie erkennen

◯ erlernte Angst z.B. vor Hunden

◯ Gewöhnung an Straßengeräusche

◯ Fahrrad fahren

FWZG1

Kreuzen Sie richtig an (1 Antwort):

Ein in einem Endknöpfchen ankommendes Aktionspotenzial öffnet dort Kanäle für…

◯ Natrium-Ionen

◯ Kalium-Ionen

◯ Chlorid-Ionen

◯ Calcium-Ionen

Instrument zum fachdidaktischen Wissen (12 Items)

FDWSK5

Begründen Sie, wieso die folgende Schüleraussage mit der Funktionsweise des Gehirns nicht vereinbar ist.

„Ich denke, dass im Kopf verschiedene Festplatten sind, die Informationen zu verschiedenen Themen gespeichert haben. Und dann, immer wenn wir ein Thema brauchen, wird eine Platte hervorgerufen.“

Ihre Antwort:

FDWSK4

Bei Schülerinnen und Schülern herrscht die Annahme vor, dass Informationen im Gehirn dauerhaft abgelegt sind und jederzeit abgerufen werden können. **Nennen Sie eine alltagsweltliche Analogie, die dieser Schülervorstellung zugrunde liegt.**

Ihre Antwort:

FDWIS7

Nennen Sie drei Schulversuche zum Thema „Lernen".

1.
2.
3.

FDWIS3

Nennen Sie eine Bedingung, die erfüllt sein muss, damit ein „Conceptual-Change" stattfindet. Beziehen Sie sich dabei grob auf die folgende Schülervorstellung.

„Ich stelle mir das Gedächtnis wie einen Computer vor, wie eine Festplatte, auf der die Informationen in einzelnen Dateien gespeichert werden."

FDWIS16

Nennen Sie eine Lernstrategiekategorie, die Schülerinnen und Schüler laut Forschungsergebnissen defizitär einsetzen und erläutern Sie, warum dies problematisch ist.

FDWIS6

Formulieren Sie für den Themenbereich „Gedächtnissysteme und Wissensarten“ <u>ein</u> Grob- und <u>ein</u> operationalisiertes Feinziel.

Grobziel:____

____

____

Feinziel:____

____

____

FDWIS12

Nennen Sie <u>zwei</u> Maßnahmen, die geeignet sind, das selbstregulierte Lernen von Schülerinnen und Schülern zu fördern.

1.____

2.____

FDWIS14

Nennen Sie zwei Methoden, mit denen Sie das Lernstrategieinventar von Schülerinnen und Schülern erheben können.

1.____

2.____

FDWIS15

Nennen Sie zwei Phasen des selbstregulierten Lernens.

1.____

2.____

FDWIS17

Nennen Sie zwei Aspekte der Methode „Struktur-Lege-Technik“, die nachhaltiges Lernen fördern.

1.____

2.____

FDWIS18

Nennen Sie zwei Aspekte der Methode „Netzwerkmethode“, die nachhaltiges Lernen fördern.

1.____

2.____

FDWIS19

Nennen Sie zwei instruktionale Unterstützungen, die als Einspeicherhilfen dienen können.

1.____

2.____

Instrument zum pädagogisch-psychologischen Wissen (17 Items)

APPW1, APPW2, APPW5

entsprechen E_D02_09_v2, E_D02_10 und E_D02_22_v2 aus Kleickmann, T., Zimmermann, F., Köller, O.; Möller, J., Hohenstein, F. (2017). Erfassung von Pädagogischem und psychologischem Wissen in der Lehramtsausbildung: Entwicklung eines Messinstruments. ZfPäd, 1, 91–113.

APPW7

adaptiert nach EW26 A aus Seifert, A., Hilligus, A.H., Schaper, N. (2009). Entwicklung und psychometrische Überprüfung eines Messinstruments zur Erfassung pädagogischer Kompetenzen in der universitären Lehrerbildung. *LbP*, 2, 82–10.

KPPW3

Geben Sie ein Beispiel dafür, wie Sie Lernstrategien in Ihrem Fachunterricht einführen bzw. fördern können.

KPPW1

Nennen Sie zwei kognitionspsychologische Modelle nach denen das menschliche Gedächtnis differenziert werden kann.

1.____

2.____

KPPW2

Nennen Sie drei kognitionspsychologische Kategorien von Lernstrategien.

1.____

2.____

3.____

KPPW4

Erläutern Sie, welche Funktion Concept Maps aus gedächtnispsychologischer Sicht haben können.

KPPW8

Kreuzen Sie richtig an (1 Antwort):

Im Experiment von Godden und Baddeley (1975) hatten Taucher Listen von Worten sowohl an Land als auch im Wasser zu lernen. Geprüft wurden sie im jeweils gleichen oder in verschiedenem Kontext. Was waren die wesentlichen Ergebnisse?

◯ Es zeigten sich keine Unterschiede zwischen den Versuchsbedingungen.
◯ Die Ergebnisse entsprachen der „Hebb´schen Lernregel“.
◯ Die Ergebnisse sprechen für die sogenannte „Enkodierungsspezifität“.
◯ Wurden die Personen in dem Kontext geprüft, in dem sie gelernt haben, war die Gedächtnisleistung wesentlich geringer.

KPPW10

Kreuzen Sie richtig an (<u>mehrere</u> Antworten):

Welche der folgenden Alternativen sind Komponenten des Arbeitsgedächtnisses?

- ◯ Zentrale Exekutive
- ◯ Semantisches Gedächtnis
- ◯ Ikonischer Speicher
- ◯ Phonologische Schleife

KPPW11

Kreuzen Sie richtig an (<u>mehrere</u> Antworten):

Welche Aussagen treffen auf die Mnemotechnik Hakenwort-Methode zu?

- ◯ Die Hakenwort-Methode eignet sich ausschließlich für das Lernen mehrstelliger Zahlen.
- ◯ Die Hakenwort-Methode verbessert die Merkleistung nur dann, wenn die Lernzeit für die Entwicklung der bildlichen Vorstellung ausreicht (mind. 4 Sekunden).
- ◯ Die Hakenwort-Methode basiert auf dem Wiederholungseffekt.
- ◯ Einer Ziffer entspricht jeweils ein bildlich vorzustellendes Objekt. Diese Objekte können miteinander kombiniert werden, um sich eine zu merkende Zahl bildlich vorstellen zu können.

KPPW12

Geben Sie ein Beispiel für Chunking.

KPPW13

Erläutern Sie die Theorie der Verarbeitungstiefe.

KPPW15

Kreuzen Sie richtig an (eine Antwort):

Welche der folgenden Alternativen ist eine elaborierende Lernstrategie?

◯ Beispiele finden

◯ Wichtiges unterstreichen

◯ wiederholt lesen

◯ meine eigenen Lernerwartungen formulieren

KPPW16

Kreuzen Sie richtig an (eine Antwort):

Welche der folgenden Alternativen ist eine metakognitive Lernstrategie?

◯ das Lernen planen

◯ Stichpunkte notieren

◯ Überschriften für Absätze bilden

◯ Arbeitsplatz aufräumen

KPPW17

Nennen Sie zwei Phasen, die beim Prozessmodell des Gedächtnisses unterschieden werden.

1.____

2.____

KPPW18

Nennen Sie ein Prinzip „gehirngerechten Lernens", das sich aus dem Prozessmodell des Gedächtnisses ableiten lässt.

____

____

Instrument zu Lernbegriffen (7 + 7 Items, 6-stufige Likertskala)

Items verwendet aus: Drechsel, B. (2001). Subjektive Lernbegriffe und Interesse am Thema Lernen bei angehenden Lehrpersonen. Münster: Waxmann.

Reproduzierender/schulischer Lernbegriff:

sich anstrengen, arbeiten, Aufgaben bearbeiten, büffeln, sich etwas einprägen, etwas beherrschen, nachdenken

Transformierender/professioneller Lernbegriff:

begreifen, beobachten, sich entwickeln, Erfahrungen machen, Problemlösen, verstehen, wachsen

Instrument zu Lernstrategien (7 + 7 Items, 6-stufige Likertskala)

Items verwendet aus: Ruffo, E. (2010). Das Lernen angehender Lehrpersonen. Eine empirische Untersuchung an der Pädagogischen Hochschule Zürich. Bern: Peter Lang.

Kognitive Lernstrategien:

Den zu lernenden Stoff gliedere ich noch einmal so, dass ich ihn verstehe.

Ich versuche immer, mögliche Alternativen zu Behauptungen und Schlussfolgerungen der Lehrveranstaltung zu finden.

Um den Lehrstoff zu strukturieren, mache ich mir oft Übersichten, Tabellen und Skizzen.

Vor Prüfungen schreibe ich zentrale Begriffe des Stoffes als Wiederholungen auf.

Wenn ich etwas gelernt habe, schreibe ich den Stoff mehrmals aus dem Gedächtnis auf.

Wenn ich etwas lernen will, mache ich mir Skizzen der wichtigsten Zusammenhänge und lerne sie dann.

Es ist immer notwendig, dass ich den gelernten Stoff mit mir bekannten Sachverhalten in Verbindung bringe.

Metakognitive Lernstrategien:

Ich stelle mir selbst Fragen, um sicherzustellen, dass ich den Stoff verstanden habe.

Wenn ich eine Aufgabe bearbeite, stelle ich mir Fragen, die mir helfen, gezielt vorzugehen.

Wenn ich ein schwieriges Problem lösen soll, passe ich mein Vorgehen den entsprechenden Anforderungen an (z.B. durch sorgfältiges Vorgehen).

Wenn beim Lernen nicht alles klappt, versuche ich, die Schwierigkeit festzustellen und die Arbeit daraufhin noch einmal durchzugehen.

Ich überlege mir während der Arbeit, ob mein bisheriges Vorgehen sinnvoll ist.

Während der Aufgabenbearbeitung überlege ich mir, ob ich mit meiner Planung hinkomme.

Wenn mir ein bestimmter Inhalt verworren und unklar erscheint, gehe ich ihn noch einmal langsam durch.

Instrument zu Überzeugungen zum Lehren und Lernen (7 + 7 Items, 4-stufige Likertskala)

Die verwendeten Items (Transmissive Überzeugungen: *IBULLKTI20, IBULLKTI24, IBULLKTI30, IBULLKTI31, IBULLKTI32, IBULLKTI36, IBULLKTI41*; Konstruktivistische Überzeugungen: *IBULLKT22, IBULLKT23, IBULLKT27, IBULLKT34, IBULLKT37, IBULLKT39, IBULLKT44* können bei den Autoren folgender Studie angefragt werden: Gimbel, K., Ziepprecht, K. & Mayer, J. (2018). Überzeugungen angehender Lehrkräfte fachspezifisch und inhaltsspezifisch operationalisieren und erfassen, in *Kohärenz in der universitären Lehrerbildung: Vernetzung von Fachwissenschaft, Fachdidaktik und Bildungswissenschaften*, hrsg. von I. Glowinski, A. Borowski, J. Gillen, S. Schanze, and J. von Meien, 179-198.

Instrument zu Überzeugungen zu Nature of Science (7 + 7 Items, 4-stufige Likertskala)

Die verwendeten Items (*IBNOSNW4, IBNOSNW9, IBNOSRB55, IBNOSRB62, IBNOSNWI23, IBNONWI22, IBNOSSV44, IBNOSRB58, IBNOSSV43, IBNOSRB57, IBNOSNWI26, IBNOSSV47, IBNOSHUI35, IBNOSSV46, IBNOSNWI25, IBNOSRB59, IBNOSNWI27, IBNOSNWI6, IBNOSNWI11, IBNOSNWI24, IBNOSRB60 IBNOSSV45, IBNOSNWI2r, IBNOSHUI37, IBNOSSV48, IBNOSNW5, IBNOSHUI36, IBNOSHUI38*) können bei den Autoren folgender Studie angefragt werden: Gimbel, K., Ziepprecht, K. & Mayer, J. (2018). Überzeugungen angehender Lehrkräfte fachspezifisch und inhaltsspezifisch operationalisieren und erfassen, in *Kohärenz in der universitären Lehrerbildung: Vernetzung von Fachwissenschaft, Fachdidaktik und Bildungswissenschaften*, hrsg. von I. Glowinski, A. Borowski, J. Gillen, S. Schanze, & J. von Meien, 179-198.
